# Evaluation of magnetic resonance spectroscopy total sodium concentration measures, and associations with microstructure and physical impairment in cervical myelopathy

**DOI:** 10.1038/s41598-025-91658-w

**Published:** 2025-02-27

**Authors:** Bhavana S. Solanky, Ferran Prados, Carmen Tur, Francesco Grussu, Selma Al-Ahmad, Xixi Yang, Alessia Bianchi, Baris Kanber, Antonino Russo, Vittorio Russo, David Choi, Jalesh N. Panicker, Claudia A. M. Gandini Wheeler-Kingshott

**Affiliations:** 1 NMR Research Unit, Queen Square MS Centre, Department of Neuroinflammation, UCL Queen Square Institute of Neurology, Faculty of Brain Sciences, University College London, Queen Square, London, WC1N 3BG UK; 2 Hawkes Institute, Department of Medical Physics and Biomedical Engineering, University College London, London, UK; 3 e-Health Center, Universitat Oberta de Catalunya, Barcelona, Spain; 4 Multiple Sclerosis Centre of Catalonia (Cemcat), Vall d’Hebron Institute of Research, Barcelona, Spain; 5 Radiomics Group, Vall d’Hebron Institute of Oncology, Vall d’Hebron Barcelona Hospital Campus, Barcelona, Spain; 6 National Hospital for Neurology and Neurosurgery, Queen Square, London, UK; 7 Department of Medicine, Surgery and Neuroscience, University of Siena, Siena, Italy; 8 Department of Uro-Neurology, The National Hospital for Neurology and Neurosurgery, Queen Square, London, UK; 9 UCL Queen Square Institute of Neurology, Faculty of Brain Sciences, University College London, London, UK; 10 Digital Neuroscience Centre, IRCCS Mondino Foundation, Pavia, Italy

**Keywords:** Spinal cord, Sodium MRS, NODDI, Cervical myelopathy, ASIA score, Macromolecular tissue volume, Predictive markers, Biomarkers

## Abstract

Spinal cord injury causes a cascade of physiological responses, which may trigger a subsequent neurotoxic increase in intracellular sodium. This can lead to neurodegeneration, both at and beyond the site of injury, causing clinical symptoms and loss of function. However, in vivo measurements of tissue sodium remain challenging. Here we utilise sodium magnetic resonance spectroscopy (^23^Na-MRS) at 3T to measure tissue sodium concentration (TSC) and its association with microstructural measures and macromolecular MRI metrics in the cervical spinal cord, distal to the site of injury. Twenty people with cervical myelopathy and twenty healthy controls, were studied. Associations with motor and sensory impairments were explored using ASIA and jOAMEQ scores. No significant difference in TSC in the cervical myelopathy group (39 ± 10 mM) relative to healthy controls (35 ± 13 mM) was found. However, patients had a significantly lower cord-cross-sectional area than controls (70 ± 9 mm^2^ vs. 82 ± 9 mm^2^, *p* < 0.001). Lower-extremity function positively correlated with intracellular volume fraction (*p* = 0.031). In conclusion, using ^23^Na-MRS, TSC in cervical myelopathy patients was successfully measured. Differences in TSC relative to healthy controls did not reach significance, despite a significant reduction in cord-cross-sectional area. However, lower intracellular volume fraction, indicating reduced neurite density distal to the site of injury, was associated with physical impairment.

## Introduction

Spinal cord injury (SCI) is a devastating event that frequently results in severe and enduring disability. Beyond the primary cause or insult in SCI, biological and pathological changes may continue, causing further, post-injury deterioration. The concept of secondary injury, for which numerous pathophysiological mechanisms have been proposed, has emerged to explain these phenomena. One explanation points towards SCI causing disruption in spinal cord blood flow leading to ischemia^1,2^. This, in turn, triggers an elevation in extracellular glutamate levels causing sodium channels to open, allowing a rapid influx of extracellular sodium ions, resulting in sodium ion retention, and thus cell death or dysfunction^3,4^.

In view of this, studies employing sodium channel blockers have been conducted with evidence that sodium channel blockers, applied within 15 min of spinal cord compression, can lead to improved outcomes pre-clinically^5^. This finding is reflected in a study on patients treated with the sodium channel blocker Riluzole within 12h of acute spinal cord injury showing improved outcomes^6^. Nonetheless, results are inconsistent throughout clinical trials and research studies^7^.

For example, a recent study in humans with cervical myelopathy, a type of SCI which involves compression of the cord, tested the efficacy of Riluzole and revealed an average gain in various clinical scores at 180 days in the Riluzole group compared with placebo, although this did not reach statistical significance^8^. Unlike the pre-clinical studies, which administered Riluzole within 12 h of injury, the cervical myelopathy patients in the study may have presented at different times post injury.

At a cellular level too, timing is key. As the intracellular sodium increases post-injury, this may induce a reverse mode of the Na+–Ca+ exchanger (NCX), increasing Ca+ influx leading to the eventual apoptosis and necrosis of cells^9^. Indeed, the timing of Na+–Ca+ exchanger interruption is relevant. In this respect, in vitro studies of anoxic injury in the optic nerve have shown a protective effect from reducing extracellular sodium concentration before anoxic injury in the optic nerve, whereas reducing extracellular sodium concentration after injury leads to more injury^10^. The disruption to the Na+–Ca+ exchanger has been shown to occur in rats, further evidenced by sensory and motor evoked potentials correlated with spinal cord blood flow^11^. These findings suggest that there is a role for sodium channel blockers in SCI, and that the timing of the injury relative to administration of Riluzole may be key in its effectiveness.

The contrasting results on the effect of sodium channel blockers in SCI studies point to a greater need for understanding dynamic sodium processes in SCI, in vivo—ideally, using a method that could be easily repeated and is non-invasive. We hypothesised that such a method could show a difference in tissue sodium and microstructural measures in the cord distal to the site of compression in CM patients relative to controls, indicating secondary injury and sodium accumulation.

Sodium Magnetic Resonance imaging offers a non-invasive, repeatable way to measure total sodium concentration (TSC) in tissue. However, its low signal-to-noise, leading to a low in plane resolution, renders it unsuitable for sodium concentration measures of the spinal cord, which has a small cross sectional area and is surrounded by CSF which is high in sodium. However, advances using sodium magnetic resonance spectroscopy (^23^Na-MRS) show that detecting changes in the TSC in the spinal cord is possible, with changes in a patient cohort of multiple sclerosis having been reported^12^. Although multiple sclerosis patients do suffer spinal cord atrophy^13^ the resultant reduction in cord cross sectional area (CSA) is not expected to be to the same degree as seen in some SCIs, for example in cervical myelopathy (CM) where the cord is compressed. The compression of the cord and disc protrusion in SCI gives rise to greater susceptibility artifacts, making MRI/MRS in this area challenging. This results in a major obstacle for robust ^23^Na-MRS measures at the site of compression, where long voxels with an excellent B_0_ homogeneity are needed. However, there is evidence that the compression site alone is not the only affected area in SCI. Indeed, microstructural changes in CM and in traumatic SCI have been reported to be present remote to the site of injury, the extent of which enables distinguishing between traumatic SCI and cervical myelopathy^14^. Atrophy affecting the cord beyond the site of injury, leading to lower cross sectional area has also been reported, again supporting that changes distal to the site of compression are present and are related to impairment^15,16^.

In view of this and the challenges of measuring TSC using ^23^Na-MRS at the site of compression, here we explore the feasibility of measuring TSC distal to the site of injury, using ^23^Na-MRS in a patient group with cervical myelopathy below C3. This patient group suffers from compression of the spinal cord affecting fine motor skills, causing pain or stiffness in the neck, a loss of balance, unsteady gait and urinary symptoms such as incontinence. In severe cases patients require surgery. Given the conservative results of sodium blockers in this patient group (an unchanged modified Japanese Orthopaedic Associations (mJOA) scores which measure the effect on neurological function, with concomitant improvements in pain scores post-surgery)^17^, the baseline levels of TSC are important to know.

TSC, as measured by MRS is a weighted sum of the intra and extracellular sodium. Changes can occur due to deviations in the intracellular and extracellular volumes or concentrations. An increase in TSC could arise due to increases in extracellular volume (due to neurodegeneration and atrophy) or an accumulation of sodium ions inside the cell (sodium retention). A decrease in TSC could, on the other hand, result from cell swelling or a reduction in serum sodium concentration, leading to hyponatremia affecting the extracellular component, which has been reported to occur in traumatic SCI^18^. To help uncouple these phenomena, diffusion weighted imaging (DWI) and normalised quantitative proton density (qPD) quantification^19,20^ can be employed. These will serve to measure tissue microstructure and macromolecular tissue volume content to establish intra and extracellular fractions and solid fractions on a voxel by voxel basis.

Whilst macrostructural and diffusion measures in cervical myelopathy have been reported the study of TSC, despite its potential importance, has been overlooked due to the challenges in its measurement. Therefore, here the TSC measured in cervical myelopathy patients using ^23^Na-MRS, distal to the site of injury, together with DWI, macromolecular tissue volume (MTV, i.e., MTV = 1 – qPD) and measures of upper cervical cross-sectional area (CSA) is interrogated and compared to values from healthy controls. The sensitivity of these MRI metrics to measures of upper and lower motor scores and function are also explored.

## Results

**Fig. 1:**
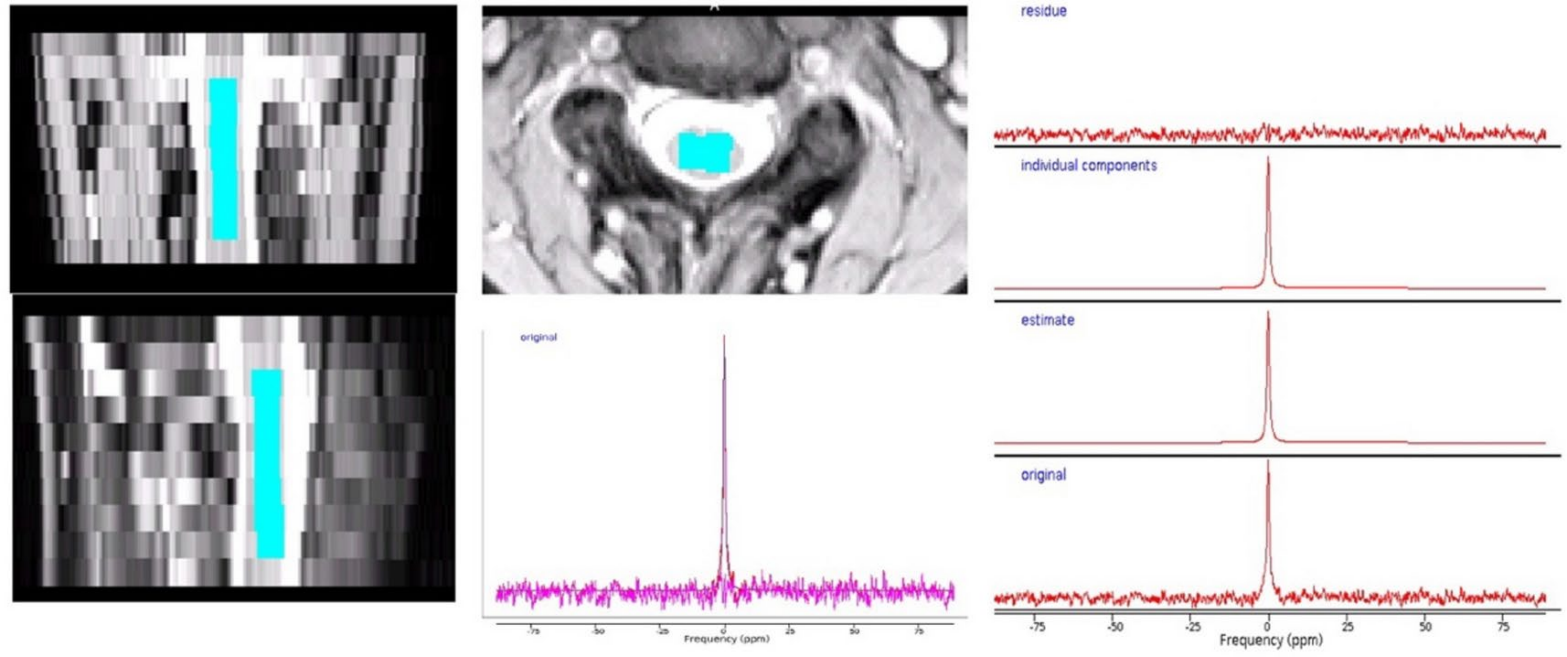
MRS Voxel placement. Example of the MRS voxel placement in the upper cervical cord and the corresponding in vivo sodium MR fitted spectrum, showing original, estimate, individual components and the residue.

Figure 1 shows the voxel placement and an example MRS spectrum of the sodium signal at the sodium resonance frequency in vivo.

**Fig. 2:**
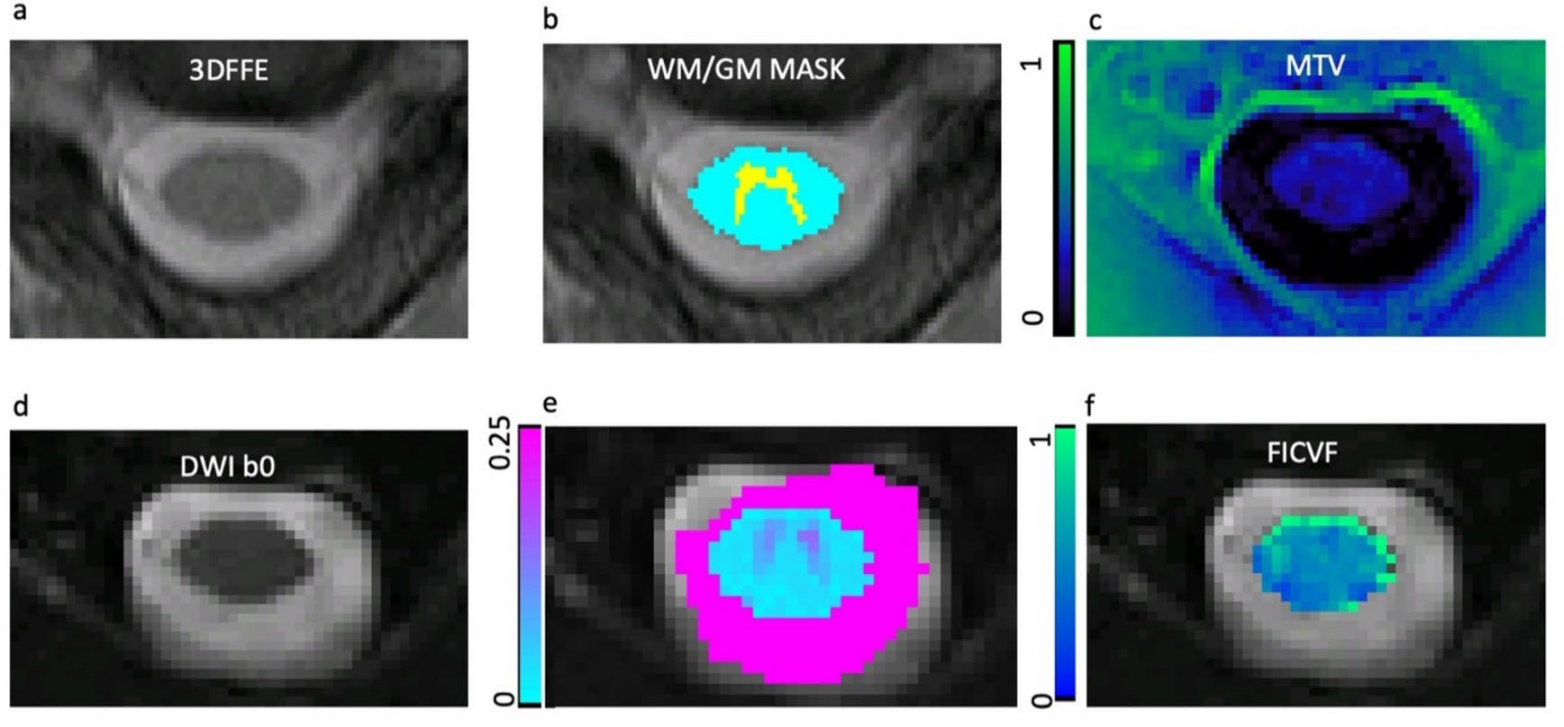
CSA segmentation, MTV and DWI maps. Example of (**a**) axial gradient echo image of the upper cervical cord, (**b**) automatic spinal cord segmentation for establishing the cross-sectional area of the upper cervical cord (WM + GM mask), (**c**) macromolecular tissue volume (MTV) map (**d**) b0 image, (**e**) orientation dispersion index (ODI) map, (**f**) intracellular volume fraction (FICVF) map.

Figure 2 shows example images for segmentation of CSA, as well as example DWI maps of intracellular volume fraction (FICVF) and extracellular volume fraction (FISO) and MTV of the solid fraction. Two subjects (one HC and one CM) did not manage to undergo sodium MRS and therefore data was not available. Further to this, ^23^Na MR spectra from n = 4 HC and n = 3 CM subjects were rejected due to poor SNR or quality (supplementary figure S2 shows examples of accepted and rejected ^23^Na-MRS spectra). As only values of FICVF, FISO and MTV were taken from the volumes corresponding to the ^23^Na MRS voxel, n = 8 DWI maps and n = 6 MTV maps were excluded, due to misregistration of the maps with the MRS voxel mask. For correlation analysis DWI data had 13 CM and 13 HC remaining, and for MTV 15 CM and 13 HC after misregistered and rejected data were removed.

### Associations with age and gender

No significant associations were found between age and MRI metrics in HC (Table 1). There was a correlation of TSC with gender in HC (Table 1). There were no other correlations between gender and MRI metrics in HC.

**Table 1:** Healthy controls association with age and gender.

	N	Unstandardised β	% 95 CI	p	R^2^
Age in healthy controls					
TSC	15	− .15	− .82 to 0.52	0.634	
CSA	16	− .04	− 1.1 to 1.0	0.941	
FISO	16	− 6.4	− 116 to 103	0.902	
FICVF	16	72	− 78 to 222	0.321	
ODI	16	− 93	− 453 to 267	0.587	
MTV	16	− 8.1	− 196 to 180	0.927	
Gender in healthy controls					
TSC	15	− 0.03	− .05 to -.02	0.001*	0.575
CSA	16	0.005	− .028 to 0.37	.755	
FISO	16	0.53	− 2.9 to 3.9	.743	
FICVF	16	3.70	− .68 to 8.1	0.092	
ODI	16	− 3.2	− 14 to 8.0	0.548	
MTV	16	1.8	− 3.9 to 7.6	0.511	

Regression analysis showing the association of age and gender on MRI metrics in healthy controls. Significant values are in italics.

*TSC = total sodium concentration, FICVF = intracellular volume fraction, FISO Fraction of ISOtropic signal, ODI = Orientation Dispersion Index, MTV = Macromolecular Tissue Volume.

### Unadjusted differences between CM and HC

Table 2 shows values for MRI metrics in HC and CM cohorts. The CM cohort has significantly lower CSA and FICVF relative to controls.

**Table 2:** Differences between cervical myelopathy and healthy controls.

MRI metric	HC	N	CM	N	Unadjusted *p* value	Adjusted *p* value
TSC (mM)	35 ± 13	15	39 ± 10	16	*0.42*	*0.53*
CSA (mm^2^)	82 ± 9	16	70 ± 9*	18	< 0.001*	< 0.001**
FICVF	0.57 ± 0.06	16	0.52 ± 0.08	16	0.047*	*0.072*
FISO	0.22 ± 0.08	16	0.19 ± 0.10	16	*0.33*	*0.26*
ODI	0.08 ± 0.03	16	0.09 ± 0.03	16	*0.23*	*0.34*
MTV	0.27 ± 0.05	16	0.24 ± 0.07	18	*0.12*	*0.15*

Group averages for total sodium concentration, microstructure (intracellular and extracellular (isotropic) volume fractions), and macromolecular tissue volume (MTV) for healthy controls (HC) and cervical myelopathy (CM) cohorts. Unadjusted *p* values, and *p* values adjusted for gender shown on the far right. Significant values are in italics.

*TSC = total sodium concentration, FICVF = intracellular volume fraction, FISO Fraction of ISOtropic signal, ODI = Orientation Dispersion Index, MTV = Macromolecular Tissue Volume, HC = healthy controls, CM = cervical myelopathy cohort. Values given as mean ± SD.

### Associations adjusted for gender and CSA

After adjusting for gender, a significant association with cohort and CSA remained (*p* = 0.001, unstandardised β = − 0.026, 95% CI [− 0.04, − 0.01], R^2^ = 0.345), while the FICVF lost significance (*p* = 0.07). Table 2 shows the values for each group and adjusted *p* values. Multiple linear regression, adjusting for gender and CSA, showed no association of MRI metrics with cohort.

### Correlations with clinical scores

**Fig. 3:**
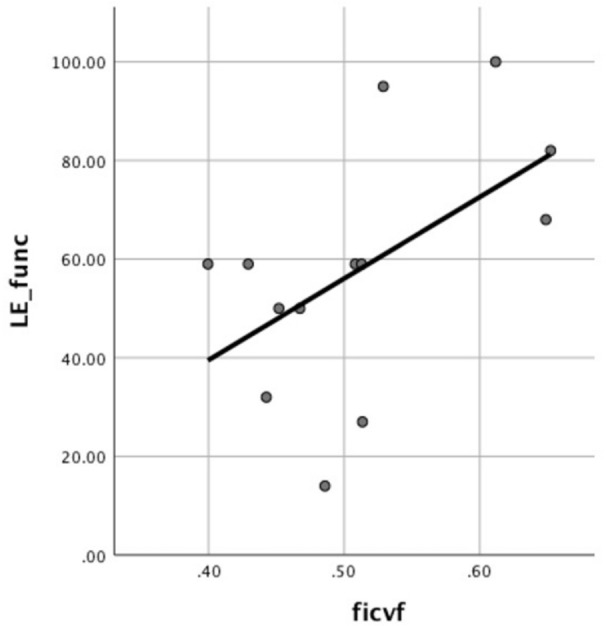
FICVF versus LE_func. Association between intracellular volume fraction (FICVF) and lower extremity function (LE_func) in the cervical myelopathy cohort.

TSC was associated with the lower extremity motor score (*p* = 0.039, unstandardised β = − 1.017, 95% CI [− 1.977, − 0.056, R^2^ = 0.269]) and FICVF was associated with the lower extremity function in CM patients (*p* = 0.031, unstandardised β = 0.002, 95% CI [0.0002, 0.003], R^2^ = 0.292), shown in Fig. 3. When adjusted for gender, the association between TSC and lower extremity motor score became insignificant, while the association between FICVF and lower extremity function remained.

## Discussion

Although average TSC in the CM cervical cord was higher than in HC, this did not reach statistical significance. This indicates that the presence of secondary injury, which leads to increased intracellular sodium, either does not occur or did not reach significance, using ^23^Na-MRS to measure TSC beyond the site of compression, in our cohort. Consequently, this small cohort alone cannot provide supporting evidence for clinical management decisions regarding the suitability or effectiveness of sodium blockers in SCI treatment, but does demonstrate that ^23^Na-MRS can be used as a tool to measure TSC in CM subjects. This work introduces a non-invasive, repeatable modality for tracking changes in TSC. The findings demonstrate the feasibility of this technique in regions distal to the site of spinal cord compression in this patient group. Given TSC changes are expected following injury, quantification of these changes using ^23^Na-MRS could be valuable for evaluating potential treatments.

It is possible that a simultaneous change in intracellular sodium (an increase) and decreased extracellular space due to cell swelling are occurring, leading to a preservation of the overall TSC, leading to a non-significant difference. However, the absence of a decrease in the FISO in this study does not support a reduction of the extracellular volume fraction. Higher resolution ^23^Na imaging, or a larger study, and models sensitive to intracellular sodium are needed to provide further evidence for the mechanisms behind these results. Moreover, higher resolution ^23^Na-MRI of the spinal cord may help assess TSC at the site of injury, in addition to exploring changes in intracellular sodium moving away from the site of injury.

Using our results a power calculation suggests that, based on the HC values of 35 ± 13 mM and an effect size of 11% (based on a mean of 39mM in the CM group), power = 0.8 and alpha = 0.05, a sample size of n = 83 would be needed. This could be reduced by using cardiac gating and boosting SNR using RF coils specific to cervical cord or higher field strengths.

It is important to note, however, that while the average TSC in the spinal cord reported here aligns with previous studies in healthy controls (HC), cardiac gating during the sodium scan was omitted, which allowed for quicker scans that were better tolerated by the patients with cervical myelopathy (CM). No cardiac gating may have impacted the sensitivity. Time from injury in the CM cohort is also an unknown, as is the case for most in vivo human studies in this population, this factor may also add variability to the results, leading to non-significant results.

Mean cross-sectional area of the cord was found to be significantly smaller in the cervical myelopathy patients relative to controls, even distal to the site of compression, supporting reports that CSA of the spinal cord is predictive of symptomatic myelopathy in degenerative cervical cord compression^21^. Given the large slice thickness needed for CSA measures in the spinal cord, and the partial volume this may introduce the sample size used was checked. Based on the healthy control CSA mean and standard deviation, power = 0.8, alpha = 0.05 and an effect size of -15%, we calculated a sample size of only n = 9 would be sufficient to demonstrate significant changes. Given our group sizes of HC = 16 and CM = 18, we can be confident of this result.

FICVF averaged over the MRS voxel was found to have no significant difference relative to controls in the CM cohort. This is in contrast to reports of reduced FICVF (neurite density) relative to healthy controls at the site of compression^22^; however, it does support studies where NODDI metrics at the site of compression were compared with those at C2/3, which were acting as control values for the CM cohort^23,24^. Larger changes in FICVF are expected at the site of compression relative to C2/3^23^, which was not explorable in this study due to the limited range of sensitivity introduced by using head RF coils in this study. No changes were observed for the orientation dispersion index (ODI) or FISO in the CM group. These results, distal to the site of injury, are also different to those at the site of injury (compression) where it is reported that ODI and FISO both differ from healthy controls^25^.

No significant changes in MTV between HC and CM were measured. Hori et al.^26^ looked at DWI (NODDI) and multi-parameter mapping to measure g ratio in cervical myelopathy, using magnetisation transfer to measure myelin volume fraction, which makes up a large portion of the solid component. They found no difference in myelin volume fraction (at C3 above the level of stenosis) relative to controls, and concluded that there was partial axonal degeneration with preserved myelin. These results support their hypothesis that any demyelination of axons as seen in preclinical studies^27,28^ has either not expanded past the site of compression in the spinal cord or that there is active remyelination, which results in MTV remaining the same as controls.

Higher FICVF correlated with a better lower extremity function score, supporting previous reports^22,29^. This finding was also supported by a study by Han et al.^29^, who found microstructural differences at C2 (distal to the site of injury) correlated with mJOA scores, which also predicted surgical outcomes. This would support function being more sensitive to the decreased neurite density, but not CSA which measures overall atrophy.

No correlations were found between MRI metrics and the more objective ASIA scores, including the lower extremity motor score. However, it must be noted that caution should be taken when interpreting values from the ASIA score in this sample, as the ASIA scores had a very limited range in the cohort (UEMS range 43–50 mean 47.5, with 8/20 scoring the upper score of 50). The narrow range of ASIA scores in the cohort is not surprising given that the ASIA scores are defined to assess traumatic SCI patients, where scores would be expected to be far lower than in CM patients, but it will impact the sensitivity of the MRI metrics to the score.

Of course limitations to the study design must be considered. In a cervical myelopathy cohort, as mentioned above the time elapsed since injury is not easily identified, as patients usually present once the injury has resulted in clinical symptoms. Therefore, the average time from injury to scan cannot be accurately determined, which may add variability to the results. Here we used only the NODDI and MTV metrics averaged over the volume corresponding to the location of the MRS voxel, namely a rectangular selection in the centre of the cord with a 35 mm length, centered at C2/3. Values therefore have been averaged across this volume, which is distal to the site of injury, and DWI and MTV weighted more heavily than other reports on the grey matter situated at the centre of the volume of interest (defined within the cord), rather than across the whole cross section. This weighting towards grey matter may have implications, as the mechanism of damage at the site of compression is known to differ between white and grey matter^10^. It is also worth noting that NODDI is optimised for white matter and hence may not be the most ideal model for GM in the spinal cord. Non-Gaussian models optimised to model GM diffusion could be explored in further studies^30^.

In addition, no measure of the magnitude of cord compression is included in our analysis, CSA is measured distal to the site of compression. The extent of compression may be a better predictor for TSC, DWI and MTV measures distal to the cord; moreover, there is evidence to suggest that changes in clinical MRI do not correlate with clinical severity^31^.

Some datasets were rejected due to misregistration of the sodium MRS voxel to the DWI and MTV space. Correlation of MRI metrics will depend heavily on the accuracy of the registration, this was visually checked for each subject to ensure accuracy but smaller less visible inconsistencies may remain. This could be minimised by obtaining all MR scans using the same RF coil, ensuring the subject remains in the same position for both proton and sodium scans. This was not utilised here as a dedicated sodium coil was used. Instead a ^1^H/^23^Na coil which gives good SNR at both frequencies would be needed.

## Conclusion

Using ^23^Na-MRS, TSC was measured for the first time in a CM cohort. Increases relative to controls did not reach significance. Clinical scores were not found to be associated to TSC or CSA. However, the intracellular volume fraction of the upper cervical cord was found to correlate to lower extremity function. This study shows that microstructural integrity, even distal to the site of compression, is related to clinical scores, but larger sample sizes are needed to detect significant differences in TSC using ^23^Na-MRS. This work provides a vital foundation for future studies on TSC measurements in SCI.

## Materials and methods

### Ethical approvals

All participants gave informed written consent to take part in the study. The study was approved by the NHS Research Ethics Service (14/NE/1006) and was performed in accordance with the Declaration of Helsinki, and in accordance with relevant the guidelines and regulations.

### Participants

Participants were twenty subjects (16 female, 4 male, 59 ± 9 years) with cervical myelopathy who attended the spinal cord clinic at the National Hospital for Neurology and Neurosurgery, Queen Square, London. The study was approved by North East—York Research Ethics Committee (14/NE/1006) and all participants gave written informed consent to take part in the study, Inclusion criteria for the CM group included awaiting Anterior Cervical Discectomy and Fusion (ACDF) surgery for de-compression at levels C3-C7. Eligible patients were within the age range 18–75 years with no other known neurological conditions or contraindication to MRI.

Cervical myelopathy in the patient population was either due to disc herniation, spondylosis or ossification of the posterior longitudinal ligament (OPLL), with cervical spondylosis being the most common cause.

The level of compression was most frequently found at the level of C5–C6 (n = 42.3%), followed by C6–C7 (n = 19.2%) and then C4–C5 and C3–C4, 23.1% and 15.4% respectively.

The most frequent symptoms patients presented with were reduced manual dexterity (clumsy hands), unsteady/impaired gait, paraesthesia and numbness of the hands and occasionally some patients reported weakness and neck or arm pain. Twenty healthy controls (10 female: 10 male, 44 ± 15 years), with no known neurological conditions, also underwent the identical MRI protocol.

### Clinical assessments

On examination patients were assessed for reflexes using tests for hyperreflexia, Hoffman’s sign, Romberg’s sign, ankle clonus and Babinski’s sign which were recorded during the MRI visit. Data were dichotomized as either being present (1) or absent (0).

The Japanese Orthopaedic Association Cervical Myelopathy Evaluation Questionnaire (JOACMEQ) was used to establish the baseline functional status of the patients. The questionnaire comprised of 24 questions which were grouped into 5 different functional domains: Cervical spine function, upper extremity function (UE_func), lower extremity function (LE_func), bladder function and quality of life. A score was computed for each factor/functional domain based on the patients’ responses (https://www.joa.or.jp/english/english_frame.html).

The American Spinal Injury Association (ASIA) score, is a standardised scale widely used for neurological classification. This was used to establish an objective measure of the patients’ motor and sensory function in SCI^32,33^. The ASIA score consists of four main domains: upper extremity motor function, lower extremity motor function, pinprick sensation PPS (tests the anterolateral/spinothalamic pathway) and light touch sensation LTS (tests the dorsal/posterior column system): ensuring that different modalities of sensation were tested which each represent different regions in the spinal cord. All these scores are measured for both the right and left side of the body separately. For the final computation of the scores, the upper extremity motor scores are added up for both sides to give a total score out of 50. The same was applied for the lower motor scores and the different modalities of sensation (maximum total of 112).

ASIA scores were measured for each patient and broken down into motor and sensory examinations. The Upper and Lower Extremity Motor score (UEMS and LEMS) and sensitivity to light-touch or pin-prick test were determined for each patient.

#### Imaging protocol

Sequence parameters are included in supplementary material Table S3.

### Sodium MR protocol

MRI scans were acquired on a 3T Ingenia system (Philips Healthcare, Best). Sodium MRS data were acquired using a fixed tuned transmit-receive sodium coil (Rapid, Germany). Using ISIS (Image-Selected In Vivo Spectroscopy), a single voxel centred on the C2-3 intervertebral disc was planned to measure sodium concentration with TR = 300 ms, effective TE = 0.26 ms, sweep width = 6000 Hz and 1024 points. Voxel size was approximately 5.8 × 7.7 × 35 mm^3^ with adjustments made per subject if this did not comfortably fit in the cord to minimise cerebrospinal fluid (CSF) contamination. Voxel length was kept fixed to 35mm to ensure registration with DWI and MTV images. Saturation bands were placed over the CSF in the anterior–posterior and right–left positions, to suppress the signal from CSF, further minimising contamination of tissue sodium signal. After scanning each subject, an identical ^23^Na-MRS scan was run on a reference phantom of similar loading containing 44.8mM sodium for calibration, using an external calibration method reported in previous studies^12^.

### Sodium quantification

The data was quantified by calibrating the amplitude of the in vivo signal with the reference phantom signal using a published method^12^. In brief data were processed using jMRUI^34^. Signal amplitudes for volunteers and phantom were measured using the AMARES algorithm. AMARES uses a non-linear-least-squares algorithm to fit the data, using prior knowledge. In this case prior knowledge was entered by picking the central frequency of the sodium peak and then defining the full width half maximum by identifying the peak at half its maximum. Differences in the performance of the ISIS sequence due to differing T1 and T2 values between phantom and tissue were also accounted for, using literature values from healthy brain tissue to correct for relaxation between RF pulses and signal acquisition, as described by previous studies^12^. Signal-to-noise > 10 was used for MRS inclusion criteria. The ratio of the two corrected signals (from phantom and in vivo), multiplied by the phantom concentration was then used to quantify the sodium concentration for each subject^12,35,36^. For registration purposes a proton axial 2D-T2w proton density scan was also acquired using the body coil before the MRS scans. This allowed the registration of a mask of the MRS voxel in sodium space to proton space.

### ^1^H MRI

Axial-oblique images, centred at C2-3 disc, were acquired using a 32ch head coil for the acquisition of images to find microstructural metrics, MTV and upper cord cross sectional area (CSA).

### Cross sectional area

^1^H gradient echo images (3D fast field echo 3DFFE), covering the same area as the MRS voxel, were acquired, using a 32-channel head coil for measurement of the cord cross-sectional area (CSA). Images were acquired in the axial plane containing 10 contiguous slices, FOV = 240 × 180 mm^2^, TR = 23 ms, TE = 5 ms, flip angle α = 7°, n = 6 with a compressed sense factor of 2 and 0.5 × 0.5 × 5 mm^3^ resolution (Table S3). This resolution is in line with other studies at 3T^37^.

CSA was computed automatically from the 3DFFE images using a spinal cord segmentation algorithm^38^, which uses an external database of spinal cord images, and their associated cord segmentations, to fit the white and grey matter in the spinal cord, providing a mask of the spinal cord for each slice. Masks of the SC were used to find the average CSA of the slices covering the ^23^Na-MRS voxel, by using the mask of the MRS voxel registered to the 3DFFE.

### Microstructure

Multi-shell diffusion weighted imaging was acquired in the cervical spinal cord, with slices covering the placement of the sodium MRS voxel. Slices were placed axial-oblique and centred on the C2/3 disc as was the ^23^Na-MRS voxel. The protocol included 68 diffusion weighted images acquired across uniformly-distributed directions (b-values: 0, 1000, 2000, 3000 s/mm^2^; δ/Δ = 20.7 ms/32.4 ms) with a reduced field of view ZOOM-EPI sequence (matrix size: 64 × 48 12 slices; resolution: 1 mm × 1 mm × 5 mm; TE = 65.5 ms; cardiac gating based on Peripheral Pulse oximetry Unit (PPU) with TR = 4RR (where RR is the time elapsed between successive R waves on the PPU device).

DWI data was then fit to the Neurite Orientation, Density and Dispersion Imaging (NODDI) model following a previously published pipeline^19^ and focussed on the following resultant indices: FISO (Fraction of ISOtropic signal coming from freely diffusing water, as in CSF or oedema), FICVF (IntraCellular volume fraction, modelled as signal from water restricted within axons and/or dendrites, hence structures approximated by sticks) and ODI (Orientation Dispersion Index, indicating variability of axon/dendrite orientation)^39^. The diffusion MRI model fitting explicitly accounts for the presence of a Rician noise floor, since an estimate of the noise level in the fitting objective function is used. This implies that the fitting routines can characterise the lack of signal in measurements taken for gradients parallel to the cord longitudinal axis for high diffusion-weighting.

Supplementary Figure S1 shows an example of images acquired for gradients roughly parallel and perpendicular to the cord longitudinal axis at b = 3000 s/mm^2^. It demonstrates that the signal has been almost completely attenuated in the former case, while it is well-above the noise floor in the latter, in line with previously reported theoretical considerations^40^.

### Macromolecular tissue volume

A previously published approach was followed^41^ based on multi-echo, variable flip angle (VFA) spoiled gradient-echo imaging^42^ to derive voxel-wise qPD estimates in the spinal cord.

qPD maps were obtained by correcting the signal for a position-dependent possible receiver bias first, as it could affect the apparent proton density (aPD) estimates from VFA T1 fitting^43^ while accounting for T2* effects. qPD was obtained by normalising aPD to the aPD of CSF and used to finally derive an index of apparent macromolecular tissue volume (MTV) content, defined as MTV = 1 – qPD. For the MTV computation, slices were acquired matching the alignment of those in DWI scans. Table S3 gives details of the multi echo VFA scans and scans for B1 mapping. MTV mapping was performed using the freely available MyRelax toolbox^20^ (https://doi.org/10.5281/zenodo.4561898).

### Registration of ^23^Na-MRS and ^1^H images

Values for DWI and MTV corresponding to the mask of the MRS voxel were evaluated for each subject. To align the sodium MRS voxel with the proton scans the 2D-T2w proton density scan was used as an intermediate reference. First, the 2D-T2w and 3D FFE images were rigidly aligned using the NiftyReg software package^44^. Next, the registration process was refined slice-wise in three steps using sct_register_multimodal from the Spinal Cord Toolbox^45^. In the first step, we registered a 3D FFE CSA mask, dilated to plus one voxel in-plane, to a mask of the cord in DWI space. This was followed by two additional steps, where the 3D FFE image was aligned with the DWI dataset using the mean b0 image. In the first step smoothed images were used for realignment, focussing on registration using broader characteristics (such as the cord outline) and the second step used unsmoothed images incorporating finer details in the registration.. Finally, all transformations were combined into a single step to map the sodium voxel mask into the DWI space. This transformation was applied using nearest-neighbour interpolation to ensure accuracy. MTV images were rigidly registered to 3D FFE space using the NiftyReg software package and the sodium voxel mask registered to the 3D FFE space was used to calculate MTV values.

Contributions from the central slices in DWI and MTV only were considered to avoid any artifacts in outer slices. Each registration was carefully checked visually for possible remaining misregistration between contrasts and compared to screenshots taken during the planning phase of the voxel to ensure accuracy of values. Subjects with erroneous masks due to intrinsic low image quality, motion or mis-registration between 3D FFE and DWI/MTV were excluded from the analysis.

### Statistical analysis

Data were analysed using SPSS (Statistical Package for the Social Sciences), IBM. Significance level was set to *p* < 0.05 for all tests.

Firstly, to check for possible confounders linear regressions were run to assess the correlation of age or gender on MRI metrics in healthy controls. Following this, the relationship of group (independent variable) and each MRI metric was run to determine the association of MRI metric and cohort. Initially an unadjusted linear regression was built to find significant associations. Following this, these associations were further interrogated using multiple linear regression to explore if the associations were still significant when adjusted for potential confounders.

To explore the association of the MRI metrics with clinical scores, an unadjusted linear regression was used, with the MRI metric as independent and the clinical score as the dependant variable. Again, these were further interrogated when using multiple linear regressions adjusted for potential confounders.

## Supplementary Information

Supplementary Information 1.

Supplementary Information 2.

Supplementary Information 3.

## Data Availability

Data is provided within the manuscript and further data is available upon reasonable request, please contact the corresponding author (b.solanky@ucl.ac.uk).

## Abbreviations

NCX: Na+–C+ ion exchanger
TSC: Total sodium concentration
CSA: Cross sectional area
CM: Cervical myelopathy
HC: Healthy controls
mJOA: Modified Japanese Orthopaedic Association scoring system
JOACMEQ: Japanese Orthopaedic Association Cervical Myelopathy Evaluation Questionnaire
DWI: Diffusion weighted imaging
qPD: Quantitative proton density
MTV: Macromolecular tissue volume
ACDF: Anterior cervical discectomy and fusion
UE_func: Upper extremity motor function
LE_func: Lower extremity motor function
ASIA: American Spinal Injury Association
UEMS: Upper extremity motor score
LEMS: Lower extremity motor score
PPS: Pin prick sensation
LTS: Light touch sensation
ISIS: Image selected in vivo spectroscopy
CSF: CerebroSpinal fluid
jMRUI: Java magnetic resonance user interface
AMARES: Advanced method for accurate, robust, and efficient spectral fitting
FFE: Fast field echo (gradient echo)
PPU: Peripheral pulse oximetry unit
NODDI: Neurite orientation, density and dispersion imaging
FISO: Fraction of ISOtropic signal
FICVF: IntraCellular Volume Fraction
ODI: Orientation Dispersion Index
VFA: Variable flip angle

## Declarations

### Additional Information

CT received honoraria from Roche, Novartis, Merck, and Bristol Myers Squibb, and is a steering committee member of the O’HAND trial and of the Consensus group on Follow-on DMTs. CGWK is a shareholder in Queen Square Analytics Ltd.
