# Supplementary material for: Evaluation of magnetic resonance spectroscopy total sodium concentration measures, and associations with microstructure and physical impairment in cervical myelopathy
Source: Sci Rep. 2025 Feb 27;15:7014. doi: 10.1038/s41598-025-91658-w (PMC11868613; doi:10.1038/s41598-025-91658-w)

Supplementary figure S1

Figure S1: Axial slices in the cervical spinal cord with diffusion gradients b=3000 s/mm^2 both perpendicular to the longitudinal axis of the cord (top), showing a lot of signal remaining and b) parallel to the cord (bottom), showing mainly noise. It demonstrates that the signal has been almost completely attenuated in the latter case, while it is well-above the noise floor in the former, in line with previously reported theoretical considerations (Grussu F et al, Magn Res Med 2019, doi: 10.1002/mrm.27463).

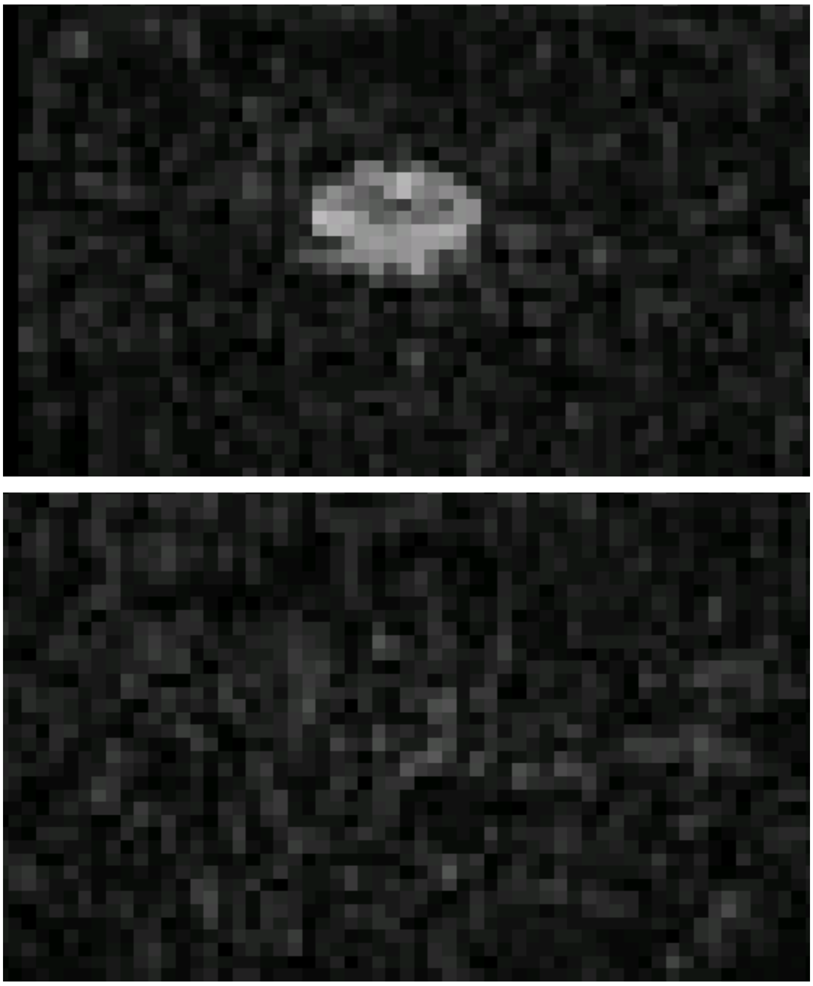

Supplement: Supplementary file 1 — Supplementary Information 1. [file 41598_2025_91658_MOESM1_ESM.pdf]
