# Supplementary material for: Evaluation of magnetic resonance spectroscopy total sodium concentration measures, and associations with microstructure and physical impairment in cervical myelopathy
Source: Sci Rep. 2025 Feb 27;15:7014. doi: 10.1038/s41598-025-91658-w (PMC11868613; doi:10.1038/s41598-025-91658-w)

Supplementary figure S2

Further spectra to show examples of rejected (top) and accepted spectra (below).

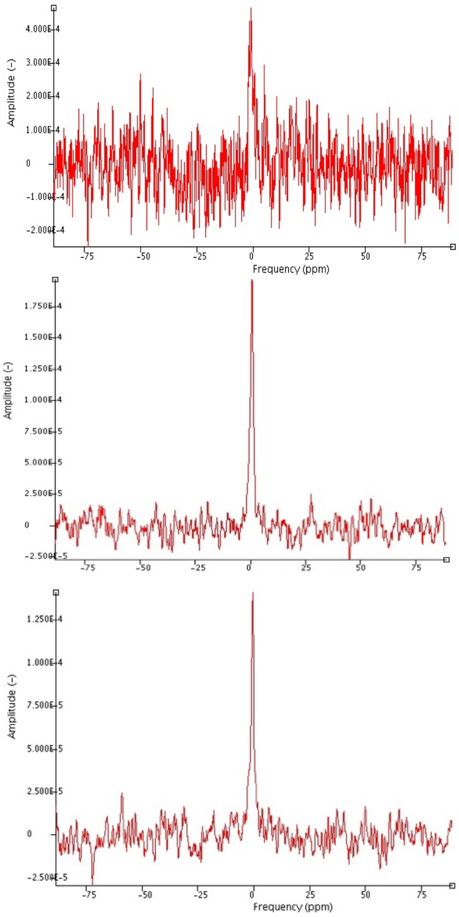

Supplement: Supplementary file 2 — Supplementary Information 2. [file 41598_2025_91658_MOESM2_ESM.pdf]
