# Supplementary material for: Evaluation of magnetic resonance spectroscopy total sodium concentration measures, and associations with microstructure and physical impairment in cervical myelopathy
Source: Sci Rep. 2025 Feb 27;15:7014. doi: 10.1038/s41598-025-91658-w (PMC11868613; doi:10.1038/s41598-025-91658-w)
Supplement: Supplementary file 3 — Supplementary Information 3. [file 41598_2025_91658_MOESM3_ESM.pdf]

Supplementary Table S3
Sequence parameters for measurement of sodium concentration, FISO, FICVF, ODI, MTV and CSA

| Sequence | Coil | TR/TE | Voxel size | FOV | NSA | specifications | Quantification |
|---|---|---|---|---|---|---|---|
| **Sodium ISIS** | T-R $^{23}Na$ (Rapid) | TR=300ms TE=0.26ms | 1.03-1.56mL ~9x12x35mm$^3$ | Single voxel centered at C2/3 | 800-1500 | Rest slabs for CSF and bone | TSC |
| **1H DWI Axial ZOOM-EPI** | 32Ch | TR=4R-R TE=65.5ms | 1x1x5mm$^3$ | 64×48mm$^3$ 12 slices | 1 | $\Delta$ = 32.4 ms, $\delta$ = 20.7 ms 64 directions at b = 1000/2000/3000 s mm$^{-2}$ Four b=0 scans | FISO, FICVF, ODI |
| **1H MTV Axial T1w 3D FFE** | 32Ch | TR=37ms TE= 2.4ms | 0.75x0.75x5mm$^3$ | 192x192x50mm$^3$ | 2 | T1 mapping: FA1=2.4 FA2=24° T2 mapping: 6 echoes $\Delta$TE=3.9ms | MTV |
| **1H AFI Axial T1w 3D FFE** | 32Ch | TR1=30ms TR2=150ms TE=2.3ms | 2.5x2.5x5mm$^3$ | 200x200x50m$^3$ | 1 | FA=60° CS Sense reduction=3 | Actual flip angle for correction in MTV |
| **1H CSA Axial 3D FFE** | 32Ch | TR=23ms TE=5.0ms | 0.5x0.5x5mm$^3$ | 192x192x50mm$^3$ | 6 | CS-Sense reduction 2 FA=7° | Cross sectional area of cord |

*TSC= total sodium concentration, CSA= cross sectional area, FICVF= intracellular volume fraction, FISO Fraction of ISOtropic signal, ODI= Orientation Dispersion Index, MTV= Macromolecular Tissue Volume
